# Exploratory Spatial Mapping of the Occurrence of Antimicrobial Resistance in *E. coli* in the Community

**DOI:** 10.3390/antibiotics2030328

**Published:** 2013-07-01

**Authors:** Sandra Galvin, Niall Bergin, Ronan Hennessy, Belinda Hanahoe, Andrew W. Murphy, Martin Cormican, Akke Vellinga

**Affiliations:** 1 Discipline of General Practice, School of Medicine, National University of Ireland, Galway, Ireland; E-Mails: n.bergin2@nuigalway.ie (N.B.); andrew.murphy@nuigalway.ie (A.W.M.); akke.vellinga@nuigalway.ie (A.V.); 2 GIS Centre, Ryan Institute, National University of Ireland, Galway, Ireland; E-Mail: ronan.hennessy@nuigalway.ie; 3 Department of Medical Microbiology, University Hospital Galway, Galway, Ireland; E-Mails: Belinda.hanahoe@hse.ie (B.H.); Martin.cormican@nuigalway.ie (M.C.); 4 Discipline of Bacteriology, School of Medicine, National University of Ireland, Galway, Ireland

**Keywords:** geographical information systems, mapping, spatial analysis, antibiotic resistance profiles, routine surveillance

## Abstract

The use of antimicrobials over the past six decades has been associated with the emergence and dissemination of antimicrobial-resistant bacteria. To explore local geographical patterns in the occurrence of acquired antimicrobial resistance (AMR), AMR of *E. coli* causing urinary tract infections (UTI) in the community in the West of Ireland was mapped. All adult patients consulting with a suspected UTI in 22 general practices in the West of Ireland over a nine-month study period were requested to supply a urine sample. Those with a laboratory confirmed *E. coli* infection were included (*n* = 752) in the study. Antimicrobial susceptibility testing was performed by standardized disc diffusion. Patient addresses were geocoded. The diameters of the zone of inhibition of growth for trimethoprim (5 μg) and ciprofloxacin (5 μg) for the relevant isolate was mapped against the patient address using ArcGIS software. A series of maps illustrating spatial distribution of AMR in the West of Ireland were generated. The spatial data demonstrated a higher proportion of isolates with AMR from urban areas. Some rural areas also showed high levels of resistant *E. coli*. Our study is the first to demonstrate the feasibility of using a geographical information system (GIS) platform for routine visual geographical analysis of AMR data in Ireland. Routine presentation of AMR data in this format may be valuable in understanding AMR trends at a local level.

## 1. Introduction

The spread of acquired antimicrobial resistance (AMR) in bacteria represents a significant public health threat, with growing limitations on the availability of effective drug treatment options for many common infections [1,2]. Studies of AMR in bacterial pathogens have emphasized changes in institutional settings, for example hospitals, or differences between institutions, regions or countries. Spatial differences in AMR at the local community level are less well described. Previous research has indicated that in addition to individual consumption of antimicrobials, geographical area-level factors also affect the risk of acquiring resistant bacteria [3,4,5]. This perspective is important, as individual use of an antimicrobial drug can impact on the risk of acquiring a resistant organism for a household and even a community [6,7,8].

The use of maps for analyzing and illustrating the occurrence of AMR in bacterial pathogens at a national level has been employed on a European-wide basis since 1999 [9]. The European Antimicrobial Resistance Surveillance Network (EARS-NET) maps show levels of resistance for key antimicrobial agents for selected species of bacteria. However EARS-NET is limited to invasive human isolates and is reported on a national, countrywide level. A similar European platform reports the variation in antimicrobial consumption in thirty countries [10]. This can help to assess relationships at a national level between the consumption of antimicrobials and the occurrence of AMR [8]. 

Differences in the levels of AMR in bacteria at the regional level have been reported in numerous countries, and practitioners are advised in many countries to consult local resistance data when prescribing antimicrobials for bacterial infections [4,5,11,12]. There are currently no platforms available for the routine analysis or reporting of AMR bacteria at the local or regional level in Ireland. Reasons for spatial variation in the occurrence of resistance at a local level can be complex, but may be attributed to differences in antimicrobial consumption [13], environmental contamination with resistant bacteria [6], localized spread through direct person-to-person transmission [14] and socioeconomic factors [15]. 

Geographical information systems (GIS) have been used as a means of conveying health care related data to a wide audience, as they facilitate the visualization of disease patterns in a specified geographic region [16]. GIS are useful in epidemiological studies as they can combine several overlaid maps to view possible associations between different factors. They are being used more frequently in primary care research [16,17]. A previous study in the West of Ireland used a GIS to investigate the spatial and temporal distribution of human cryptosporidiosis over a three-year period and to highlight areas and seasons of high risk [18]. A major outbreak of cryptosporidiosis occurred during this period and the resulting GIS information was helpful in analyzing and presenting data on the outbreak. GIS have also been implemented for the study of other infectious diseases in Ireland, including a longitudinal study of bovine tuberculosis [19].

Investigations into the spread of resistant pathogens in the wider community represent a major challenge for public health. Community based investigations generally involve broad geographical regions, and there are currently no established regional AMR reporting systems in place in Ireland. The appropriate medium would need to convey relevant health information, such as resistance data, to health care professionals at all levels, in particular general practitioners, on a routine basis. The ideal platform for this type of data would give an easy to understand, clear and concise overview of bacterial AMR on local, regional and national levels. This platform should also support analysis of spatial variation in the occurrence of AMR in the context of spatial data on antimicrobial consumption and other health indicators (e.g., deprivation indices, land use patterns). This information would promote health care professionals’ and communities’ awareness of the local occurrence of resistance and ultimately to understand to what extent factors such as local antimicrobial consumption impact on local resistance levels. The routine provision of AMR data in the primary care setting in this format could support a positive change in antimicrobial prescribing practices, as prescribing would be guided by local resistance patterns. 

GIS are currently underutilized for studying AMR in the community and have not been used previously for this purpose in Ireland. Previous research elsewhere applied GIS to study meticillin-resistant *Staphylococcus aureus* (MRSA) [20], *Streptococcus pneumoniae* [21] and *E. coli* [22]. The present study aims to use GIS to present AMR to two important therapeutic agents in *E. coli* causing UTI from a prospective study in a region in the West of Ireland, details of which have been previously reported [23]. The agents studied were trimethoprim, which is currently recommended as first line treatment for UTI in Ireland, and ciprofloxacin, a fluoroquinolone, recommended as a reserve second line agent [11]. Antimicrobial susceptibility data from all *E. coli* isolates associated with UTI in the community during the study period were mapped against the geo-coordinates of the patients. Since Ireland does not have postal codes, geocoding was done by hand, based on the townland address. This process aided a feasibility assessment for the routine use of GIS in analyzing and reporting AMR information. 

## 2. Results

### 2.1. Descriptives

The current study included 682 females (91%). The overall patient mean age was 55 (±21) years (Table 1). Overall, female patients were younger compared to males (55 ± 21 compared to 64 ± 18 years).

**Table 1:** Summary of patient descriptive.

	N	%
Female	682	91%
Nursing home residents	70	9%
	**mean**	**SD**
Age	56	(±21)

### 2.2. Distribution of UTI Cases

**Figure 1:**
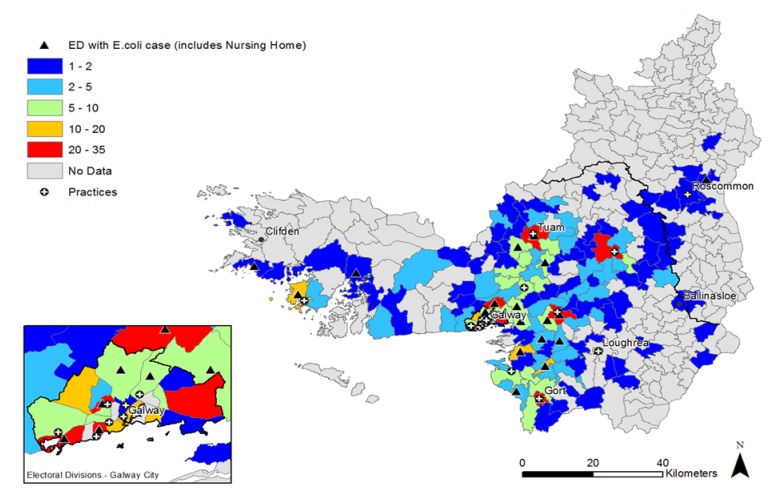
Distribution and frequency of *E. coli* urinary tract infection (UTI) cases, practices and nursing homes in the study region at electoral division level.

The distribution of *E. coli* UTI cases, study practices and nursing homes in the study region are outlined in Figure 1. A high number (*n* ≥ 10) of *E. coli* cases per electoral divisions (ED) are indicated with a red or orange color. A low number (*n* ≤ 5) of cases are indicated with blue or green. Practices and nursing home locations are identified in each ED. High numbers of UTI cases are observed in more urban areas with a higher population density. 

### 2.3. Antimicrobial Resistance Patterns

**Figure 2:**
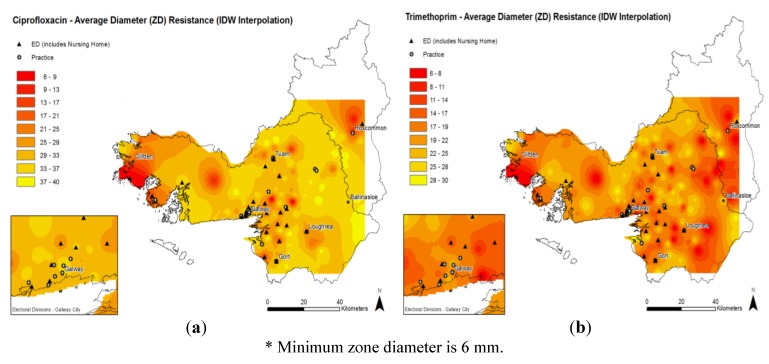
Average antimicrobial susceptibility results * for *E. coli* isolates for (**a**) ciprofloxacin (left) and (**b**) trimethoprim (right).

Heat maps showing the mean antimicrobial diameter of the zone of inhibition results for *E. coli* to ciprofloxacin and trimethoprim are presented in Figure 2a,b. These maps display results without the differentiation of the individual ED from where the isolates were obtained. The results are not interpreted according to any breakpoint criteria. Darker regions correspond to localities where isolates with lower zone of inhibition diameters, indicating higher levels of resistance to the antimicrobial agent, were obtained. The heat maps indicate that for both trimethoprim and ciprofloxacin, higher levels of antimicrobial resistance (lower zone diameters) seem to occur in urban areas. Higher levels of resistance were also noted in areas where isolates were obtained from patients in nursing homes, however this result is tentative given the low number of isolates included in the study. While resistance to trimethoprim is more widely disseminated in the region, similar hotspots of resistance for both ciprofloxacin and trimethoprim can be observed in both maps, in particular towards the extreme western and eastern regions of the maps. 

**Figure 3:**
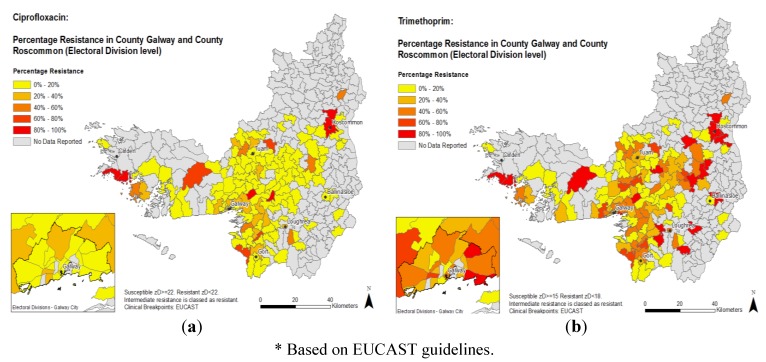
Percentage antimicrobial resistance* for *E. coli* isolates for (**a**) ciprofloxacin (left) and (**b**) trimethoprim (right) based on European Committee for Antimicrobial Susceptibility Testing (EUCAST) guidelines.

A choropleth map (Figure 3a,b) displays the proportions of trimethoprim and ciprofloxacin resistance in each individual ED according to the current European Committee for Antimicrobial Susceptibility Testing (EUCAST). This map also shows similar hotspots of AMR as noted in the scalar format in Figure 2. 

## 3. Discussion

The present study demonstrates the feasibility of the application of GIS for studying the dissemination of acquired AMR in a common bacterial pathogen in the wider community at a local level. GIS software allows for the analysis and visual representation of data in the form of a geographical map, which can be easily interpreted by health care professionals, members of the public and policy makers [16]. Results should be considered in the context that the limited number of *E. coli* UTI isolates occurring in many individual EDs contributes to potential differences in AMR that occur by chance. Nevertheless, the results of the current study suggest spatial clustering of resistance and potential areas of high-risk for antimicrobial-resistant *E. coli*.

The study uses both clinically interpreted (EUCAST) and non-interpreted (zone diameter) results to present the data, which also allows for the visualization of a summative quantitative measure of resistance (mean zone diameter) in addition to the more commonly used percentage of isolates categorized as resistant by defined interpretive breakpoint criteria. 

The results suggest spatial clustering of resistance to trimethoprim and ciprofloxacin and therefore the possibility of local high-risk areas of resistant bacteria. The use of GIS allows one to examine spatial variations at the level of resistance in the context of particular geographical information, for example urban compared with rural areas or the occurrence at a nursing home in the locality. There is a subjective impression, for example, of a link between higher resistance among *E. coli* in a locality and the presence of a nursing home in the area, although this is both unconfirmed and difficult to interpret. The inclusion of isolates from nursing homes in the current study introduced bias. UTIs are very common in nursing home residents and urine samples are regularly taken to check for infection. A higher proportion of UTIs is therefore to be expected in areas with a nursing home. The proportion of resistant *E. coli* UTIs is also higher due to a combination of high antimicrobial consumption by the residents and interpersonal spread. The inclusion of isolates from nursing homes will inflate the overall AMR level of a particular area compared to townlands where no nursing home is present, in particular with the low numbers included in this study. Previous research has identified household and community risk factors for the acquisition of resistant bacterial pathogens. The role of the wider environment in the spread of AMR is now better understood [6]. A study investigating the geographic risk associated with community acquired MRSA in the US identified a significant cluster of MRSA isolates (*n* = 27) from patients within a specific area of a city [20]. The authors suggest that these maps be used to advise antimicrobial therapy for individuals within the region and that the patients address should be taken into consideration when appropriate empirical treatment options are being considered. 

The use of GIS to visually present the association between antimicrobial consumption and antimicrobial prescribing was previously carried out in Sao Paulo, Brazil [22]. This previous study focused on ciprofloxacin resistance in *E. coli* as a urinary tract pathogen and correlated high-level resistance with antimicrobial consumption expressed as defined daily doses (DDD). Similar to the current study, clustered hot-spots of resistance were observed along with significant spatial variation. Resistance hot spots correlated to higher usage (5–9 DDDs per 1,000 inhabitants) of ciprofloxacin in the community. General practitioners (GPs) could benefit from the routine use of GIS for displaying AMR data by aiding in the prescription choice of antimicrobial drugs for common infections. The maps could indicate if the GP is in an area where high or low resistance to certain drugs is prevalent in the bacterial population. This would result in less use of newer, reserve antimicrobials (particularly in areas of low resistance), hence more appropriate and better treatment outcomes for patients (particularly in areas of high resistance). Conversely, GIS could be used to show antimicrobial prescribing patterns as a direct measure of antimicrobial resistance in a specified region; however this was not carried out in the present study. 

While there are numerous advantages of using GIS to display health care information, appropriate precautions must be adhered to. When inferring a relationship based on the results of spatial analysis, caution should be used in applying the appropriate areal units (scale of the map) and analysis should be carried out at numerous scales to evaluate the effect [16]. The use of the Kernel function to generate heatmaps is sensitive to any bias that may be present in the original data used. The patient data used in the current study was limited to the patient address at the townland level to ensure that specific patients could not be identified and for feasibility of use. However there is a potential risk of bias due to the aggregation of *E. coli* information at the townland level. This is particularly apparent with the inclusion of isolates from nursing home residents, where a greater number of *E. coli* isolates with increased AMR can inflate the observed levels of resistance in that area, as previously discussed.

The use of GIS for mapping health care data based on patient addresses in Ireland on a routine basis has several limitations. Geocoding of patient addresses can be a time-consuming task, in particular where postal codes are not in use, as is the case in Ireland. Numerous discrepancies exist between the spellings of various townlands, in particular where Irish is the first language (Gaelic-speaking areas). The introduction of postal codes in Ireland, or the automatic linking of patient addresses with x, y coordinates, would alleviate this problem and allow for automated patient data retrieval and subsequent analysis. 

This is the first study to report the use of GIS for the investigation of patterns of antimicrobial resistance in pathogenic bacteria in Ireland. The study highlights the potential of GIS to present antimicrobial resistance data for routine communication of current epidemiological trends in bacterial pathogens. As piloted in this study, this can allow the investigation into the impact of possible risk factors associated with antimicrobial resistance, such as the presence of a health care center such as a nursing home in the region. While not evaluated in the current study, the software can also be used to investigate the impact of other potential risk factors such as social deprivation or agricultural use in the region [18], as well as correlating antimicrobial prescribing data with resistance patterns, which is currently being analyzed in a separate study. 

## 4. Experimental

### 4.1. Patient Selection

The study area covered two counties in the West of Ireland, namely Galway and Roscommon (total area = 8,695 Km^2^). Patient data from a previous prospective case-control study analyzing AMR (trimethoprim and ciprofloxacin) in *E. coli* causing UTI in the community was used [3]. Details of ethical approval, practice selection and patient inclusion criteria are described in previous papers [3,24]. The previous prospective study was carried out over a nine-month period in 22 participating practices. All adult patients with a suspected UTI were requested to supply a urine sample for analysis at the regional diagnostic laboratory. A total of 778 patient records from 22 practices in the West of Ireland were selected. Of these patients, 23 were excluded because the patients lived outside the geographical area being studied. A further three were excluded on the basis of having incomplete data, giving a final sample size of 752 patients (Table 1). 

### 4.2. Data Collection

To obtain patient addresses at the townland level, the results from the patients’ analyzed urine samples were accessed at the University Hospital Galway (UHG). Antimicrobial susceptibility zone diameters (mm) to two antimicrobials (trimethoprim and ciprofloxacin) and patient townland addresses were geocoded with assignment of specific latitude and longitude coordinates, for use with ArcGIS software as described below.

### 4.3. Geocoding, Mapping and Cartographic Displays

Patient townland data was converted to a vector point Shapefile by extracting the centroid (x,y location) of each townland polygon. Following this, a spatial join was carried out in ArcGIS to link the patient data with the geographic townland dataset. Electoral divisions (ED) (*n* = 342) were used as base scale features (polygons). EDs are the smallest legally defined administrative areas in Ireland, of which there are 3,440 [25]. The resultant dataset provided a geolocated townland and electoral division address for each patient. Each of the practices (*n* = 22) was manually geolocated on a base map in ArcGIS. 

Antimicrobial susceptibility testing was carried out using the disk diffusion method of the European Committee on Antimicrobial Susceptibility (EUCAST) [26]. The diameter of the zone of inhibition (mm) of growth was mapped against patient addresses and zone diameters were interpreted (resistant or susceptible) according to the EUCAST clinical breakpoints and were also mapped [26]. The minimum zone diameter that can be obtained is 6 mm (diameter of antimicrobial disk used in the test). 

Color themed maps were created to visually display the number of UTI cases per ED. Where an isolate originated from a nursing home patient, the nursing home was indicated in an ED. Heatmaps were generated by extracting the average zone diameter for each ED, while choropleth maps were generated using proportions of resistance for each ED. Heatmaps use varying degrees of color (yellow, orange, red) to represent a scale. In this study, a lighter yellow color indicates a larger zone diameter result and less resistance to the specific antimicrobial studied. A deeper orange color indicates a smaller zone diameter result indicating increased resistance to an antimicrobial, while a red color indicates complete resistance (*i.e.*, a minimum zone diameter of 6 mm) to the antimicrobial. 

The spatial extent of heatmaps (Figure 2a,b) does not extend to the boundary of the two study area counties. This is a factor of the distribution of the sample locations. In an effort to convey accurate information pertaining to AMR within the study areas, regions that do not have corresponding AMR data have been left blank (no data).

## 5. Conclusions

This research demonstrates that it is possible to spatially analyze community antimicrobial resistance in common bacterial pathogens. This suggests a potential application of geo-mapping to optimize antimicrobial prescribing practices across large geographical regions. 
